# NK cell predicts the severity of acute graft-versus-host disease in patients after allogeneic stem cell transplantation using antithymocyte globulin (ATG) in pretreatment scheme

**DOI:** 10.1186/s12865-019-0326-8

**Published:** 2019-12-09

**Authors:** Ping Zhang, Shujun Yang, Yujing Zou, Xiao Yan, Hao Wu, Miao Zhou, Yong Cheng Sun, Yi Zhang, Huiling Zhu, Kaihong Xu, Yi Wang, Li Xia Sheng, Qitian Mu, Liguang Sun, Guifang Ouyang

**Affiliations:** 1 Department of Hematology, Ningbo First Hospital, Ningbo, 315010 China; 2 Laboratory of Stem Cell Transplantation, Ningbo First Hospital, Ningbo, 315010 China; 3 Key Laboratory of Organ Regeneration & Transplantation of the Ministry of Education, The First Hospital of Jilin University, Changchun, China; 4 Division of Hematologic Malignancies and Cellular Therapy, Duke University Medical Center, Durham, NC 27710 USA

**Keywords:** Graft-versus-host disease, Antitymocyte globulin, NK cells, stem cell transplantation

## Abstract

**Background:**

Graft-versus-host disease (GVHD) is one of the most complex complications after allogeneic stem cell transplantation. Current standard of grading system is based on clinical symptoms in skin, liver and intestinal. However, it’s difficult to differ GVHD and its extent just by clinical manifestation. Here we retrospectively analyzed cell immune function in patients implemented allogeneic stem cell transplantation in Ningbo first Hospital from Jan 2013 to Jan 2018.

**Results:**

the data are collected from 51 patients (mean age was 42; 45.1% women). The average NK cell percentage was 39.31% in severe GVHD (Grade III-IV), was 16.98% in mild GVHD (GradeI-II), while was 21.15% in No GVHD group. The statistical analysis showed difference among each grade. Further analysis was performed in Antithymocyte globulin (ATG) treated group and control group. We showed NK Cell percentage was sharply different in ATG treated group: 47.34% in severe GVHD, 11.98% in mild GVHD group, while 18.3% in no GVHD group. However, in control group, the average percentage of NK cells was 23.27% in severe GVHD, was 23.22%in mild GVHD group, while was 21.13% in no GVHD group.

**Conclusion:**

The data supports that ATG can prevent GVHD by increasing NK cell percentage. The percentage of NK cell seemed to be a useful probe to evaluate the severity of GVHD in allogeneic stem cell transplantation patients using ATG in pretreatment.

## Background

Graft-versus-host disease (GVHD) poses as a major complication following allo-genetic hematopoietic stem cell transplantation (allo-HCT). GVHD occurs in both acute and chronic forms, which can lead to morbidity and mortality [1]. Allo-reactive donor T cells, which are the primary mediator of GVHD, can secret multiple cytokines and initiate cytokine storm [2]. According to classic standards, acute GVHD can be divided into 4 different grades depending on the degree of damage to the skin, liver, and gastrointestinal tract. Although grades 3 and 4 are considered to be severe GVHD according to the criteria due to the delay clinical manifestations or the interrupt of treatment. By the same token, a 1–2 degrees GVHD can be fatal if not immediately treated. Therefore, the time of intervention is critical particularly for patients may develop lethal GVHD. However, there is currently a lack of understanding in this field. While researchers attempt to distinguish between severe and non-severe GVHD through clinical manifestations, there is a lack of effective detection methods to determine the critical point of intervention in order to prevent disease development as early as possible for lethal GVHD.

Antithymocyte globulin (ATG) is a polyclonal antibody against fresh human thymocytes derived from rabbits, horses, or pigs. It has been used as a T cell-depleting agent in stem cell transplantation and organ transplantation, and has been found to decrease the incidence of GVHD [3]. Due to its polyclonal nature, it is possible that it may be able to recognize targets beyond T cells alone. ATG can influence intracellular interactions and regulate lymphocyte cytokine production through different mechanisms. A multicenter clinical trial investigated rabbit-derived ATG(rATG) function in acute leukemia patients who received peripheral blood stem cell transplantation from HLA matched siblings. The study revealed that the use of ATG as a myeloablative conditioning regimen was able to decrease the risk of chronic GVHD [4]. The incidence of GVHD has increased as more patients undergo haploidentical stem cell transplantation. The use of ATG may affect the microenvironment by suppression of pathogenic T cells as well as promoting immune reconstitution (IR) including T cell subsets [5]. Former studies suggest that Regulatory T cells (Tregs) can enhance recovery of a broad T-cell repertoire [6] to promote immune reconstitution and prevent graft-versus-host disease (GVHD) after hematopoietic stem cell transplantation [7]. NK cells play as an immune surveillance role in malignant hematology disease, study proved it can eliminate leukemic cells, restore graft-versus-leukemia function in allogeneic stem cell transplant, and induce minimal graft versus host disease [8]. The protective function in GVHD may because of the KIR-ligand mismatch [9].

The use of ATG may alter the immune cell repertoire in vivo sharply, which may provide clues for the prediction GVHD development and severity. Although the criteria for the clinical manifestations of GVHD, it remains difficult to predict the severity of GVHD in some cases. We speculate that the microenvironment of the graft recipient may vary with the use of ATG, resulting in variations in the onset and degree of severity in GVHD. It may therefore be possible to predict GVHD by monitoring changes in immune cell subsets after transplantation preceded by the use of ATG as part of the myeloabaltive regimen.

Previous studies have found that the frequency of Tregs is inversely correlated with GVHD development [7, 10] while the infusion of exogenous NKT cells can reduce the degree of GVHD. Former study showed that while naïve T cells (CD44^lo^CD62^hi^ in mice, CD44^low^CCR7^high^CD45RA in human) can induce GVHD, effector T cells (CD44^high^CD62L^lo^ in mice or CD44^lo^CCR7^hi^CD45RO in human) do not induce GVHD, which could be explained by memory T cell apoptosis or exhaustion following alloantigen exposure [11, 12]. The relevant findings were based on myeloablative conditioning regimens in models that involve total body irradiation or chemotherapy. However, none included ATG treatment, a procedure widely used in the clinic prior to haplo-identical allogeneic hematopoietic stem cell transplantation (allo-HSCT). Whether the incidence and development of GVHD would be affected due to the use of ATG in the preconditioning regimen remains unknown. Furthermore, it is also unclear if a correlation exists between GVHD and variations in immune cell subset frequency in the context of ATG treatment.

As one of the major causes of mortality after allo-HSCT aside from relapse of the primary disease, GVHD has been found to be promoted by T cell-induced cytokine storm. Although total T cell number can be recovered approximately 3 months after allo-HSCT, the recovery of CD4 helper T cells (T_h_ cells) requires more than 1 year [5]. Consequently, the T_h_ cell deficiency renders patients susceptible to infection aside from the concurrent lack of protective immunoglobulins. Furthermore, the rapid recovery of donor-derived alloreactive T cells significantly increases the incidence of GVHD [11]. Removal of T cells using ATG can alleviate T cell-mediated immune attacks, thereby reducing the incidence of GVHD [4], the incidence of cGVHD decreased from 69 to 32% [3]. However, even with the use of ATG in haplo-identical stem cell transplantation, the probability of the procedure causing GVHD is higher than identical stem cell transplantation due to the source of stem cell. ATG can inhibit T cell proliferation, primarily targeting CD8 T cell rather than CD4 T cells, due to the higher percentage and rapid expansion of the former [13]. In addition, although various lymphocytes have been shown to be directly or inversely corelated with GVHD development, including memory T cells, Treg cells, Th17 cells, NK cells, the severity of GVHD can be difficult to predict in vivo with the use of ATG. It is unclear whether the corresponding lymphocyte subsets remain the same in patients that do not receive ATG. Here we attempt to address the above questions to better predict the severity of GVHD development.

In this study, we analyzed 51 adult patients who received allogeneic hematopoietic stem cell transplantation (HSCT) with or without ATG treatment. We recorded the changes of immune cell subsets in the recipients after the use of ATG, and analyzed the correlation between these changes and the occurrence and severity of GVHD. Specifically, focused on NK cells and CD4^+^CD25^+^ T cells in ATG-treated patients who underwent allo-genic stem cell transplantation to elucidate the underlying mechanisms of the suppressive activity of ATG.

## Methods

### Patients and sample collection

This study enrolled 51 patients (17-68 years) diagnosed with acute myeloid leukemia(AML)/acute lymphocyte leukemia(ALL)/myelodysplastic syndrome(MDS)/chronic myeloid leukemia(CML)/aplastic anemia(AA)/ paroxysmal nocturnal hemoglobinuria(PNH) who received allogeneic hematopoietic stem cell transplantation in Ningbo First Hospital between Jan 2013 to Jan 2018 (Table 1), patients with hematologic malignancies or non-malignant diseases including aplastic anemia and PNH were included. Among all patients, 27 received stem cell transplant from either HLA-identical related donor (22 patients including 1 patient received 9/10 HLA identical donor) or HLA-identical unrelated donor (5 patients), while the remaining 24 patients received stem cell transplant haplo-identical donors. GVHD prophylaxis consisted of cyclosporine, mycophenolate mofetil (MMF) (half dose after neutrophil granulocyte engraftment), and intravenous methotrexate (15 mg/m2 on day + 1, then 10 mg/m2 on days + 3, + 6 and + 11). Furthermore, 30 patients were administered with ATG using conditioning treatment for GVHD prophylaxis, including 5 AA patients and 25 patients who received stem cells from haplo-donors. The other 21 patients did not receive ATG treatment. Immune reconstitution of CD4, CD8, NK cell, CD4 + CD25 + T cells, and CD8 + CD38+ cells was monitored for 1- to 2- year period. Exclusion criteria included engraft failed in the first month after transplantation, severe infection during transplantation. Fresh blood sample were obtained at the beginning of each month after transplantation and 1–3 months before the development of GVHD. Written informed consent was obtained from all patients prior to sample collection, in accordance with the Declaration of Helsinki. Protocol approval was obtained from the Human Subjects Protection Committee of Ningbo First hospital.

**Table 1:** Patient characteristics

	All cohort	Severe GVHD (grade III-IV)	Mild GVHD (grade I-II)	No GVHD	
	51	9	18	24	
Male sex	28	5(17.9)	10(35.7)	13(46.4)	
Conditional regimen					*P* = 0.86
With ATG used	30	6(20)	10(33.3)	14(46.7)	
No ATG used	21	3(14.3)	8(38.1)	10(47.6)	
Age	42(17–68)	50(17–67)	42(25–68)	42(17–67)	*P* = 0.7
Diagnosis					*P* = 0.14
Acute leukemia+MDS	33	5(15.2%)	16(48.5%)	12(36.4%)	
Chronic myeloblastic leukemia	5	2(40%)	2(40%)	1(20%)	
Lymphoma+MM		1(16.7%)	0(0%)	5(83.3%)	
Aplastic anemia+PNH		1(14.3%)	0(0%)	6(85.7%)	
Donor HLA type					*P* = 0.49
Haplo	24	5(20.8%)	10(41.7%)	9(37.5%)	
Sibling(9/10,10/10)	22	4(18.2%)	7(31.8%)	11(50%)	
MMUD	5	0(0%)	1(20%)	4(80%)	
Stem cell source					*P* = 0.44
PBSC	27	4(14.8%)	8(29.6%)	15(55.6%)	
PBSC+BM	24	5(20.8%)	10(41.7%)	9(37.5%)	
Conditioning regimen					*P* = 0.75
Myeloablative conditioning	46	8(17.4%)	17(37.0%)	1(45.7%)	
RIC	5	1(0%)	1(80%)	3(20%)	

### Flow cytometry

50ul of blood samples including 1 × 10^6^ mononuclear cells were incubated with titrated antibodies for 15 min at room temperature in the dark. RBCs were lysed with FACS lysing solution (BD Biosciences, San Jose, CA). Cells were stained with antibody cocktail and analyzed using a FASCanto flow cytometer equipped with FACSDiva software (BD Biosciences). Absolute cell counts were determined using Flow-Count Fluorospheres (Beckman Coulter, Brea, CA). The antibodies included FITC-conjugated anti-CD62L (clone MEL-14), R-PE–conjugated anti-CD45.1 (clone A20), cy-chrome-conjugated anti-CD44 (clone IM7), and the corresponding isotype controls (all from BD PharMingen); FITC-conjugated anti–H-2Db (clone CTDb), PE-conjugated anti-CD4 (clone CT-CD4), B220 (clone RA3-6B2), PE-Texas red–conjugated anti-CD4 (clone RM4–5), tricolor-conjugated anti-CD4 (clone CT-CD4), CD8a (clone CT-CD8a), and corresponding isotype controls (Invitrogen, Carlsbad, CA). After each graft collection, samples were stained using the multitest kit (Becton Dickinson, Le Pont de Claix, France). A panel of directly conjugated monoclonal antibodies (Mabs) was used to separate distinct T-cell subsets and homeostatic characteristics of each subset. Activated CD4 T and NK cells were defined as CD4 + CD25+, CD3-CD56 + CD16+, respectively. The panel design included: CD3FITC/CD56CD16PE/CD19APC/CD45PE-Cy7; CD3FITC/CD25PE/CD4PerCP-Cy5.5/CD45PE-Cy7 and CD38FITC/CD8PE/HLADR PerCP-Cy5.5/CD3APC/CD45PE-Cy7 (Additional file 1: Figure S1).

### Statistics

Patient baseline characteristics and immunophenotype data were analyzed. T cell subsets data were compared using Student’s t-test for unpaired group comparison and the Wilcoxon signed-rank test for paired comparison. All tests are 2-sided at the significance level of 0.05 and multiple comparisons were not included. All statistical analyses were performed using SPSS version 17.0. All graphs were made using Prism software (GraphPad).

## Results

### Patient characteristics and treatment outcomes

A total of 51 patients were analyzed in current study. The median age was 42 years (range 17–68 years), 86.3% (44/51) had hematologic malignancies (MDS 15, AML 14, ALL 4, CML 5, NHL 4, MM 2), 13.7% (7/51) were diagnosed with nonhematologic malignancies, which including 5 AA patients and 2 PNH patients. Myeloablative conditioning was performed in 46 patients (90.2%), peripheral blood stem cells were used as the only source of stem cell in 27 patients (52.9%). Furthermore, 27 patients (52.9%) underwent transplantation from HLA-matched donors, including 22 from siblings, 5 from the bone marrow bank, while the remaining 24 patients (47.1%) received haplo-matched transplantation. Main clinical characteristics of the patients included in the study were summarized in Table 1.

### Conditioning regimen and transplant procedure

Among HLA-matched sibling and 5 AA transplant recipients in the ATG-treated group (all stem cells were derived from the bone marrow bank, one patient with 9/10 matched donor), all patients received a small dose (2.5 mg/kg) of ATG in the conditioning regimen. In addition, all patients in the haplo-HSCT transplantation group received ATG in the pretreatment. Furthermore, 5 patients received the reduced dose regimen while the remaining 46 patients received busultfan/cyclophosphamide (BUCY, 12/51) or modified BUCY (34/51). Filgrastim-mobilized peripheral blood stem cells. All 51 patients reached complete chimerism by day 30. The median neutrophil cell recoverytime was 13 days (10–30) while the median platelet recovery time was 15 days (9–221).

### GVHD prophosis and T cell function analysis

In all patients, GVHD prophylaxis included cyclosporine, mycophenolate mofetil, and methotrexate. For GVHD treatment, prednisone or methylprednisolone were added. For severe GVHD, CD25 or anti-IFN-α antibody were used. In retrospective analysis, GVHD were graded using the classic criteria. Severe GVHD were defined as grade III and IV while mild GVHD were defined as grade I and II. In addition, due to the uncertainty in the GVHD grading for lethal versus non-lethal GVHD (i.e. in some cases, the symptoms during the early stage of disease GVHD performance could not be definitively diagnosed), the grading of GVHD was corrected in the retrospective analysis.

**Fig. 1:**
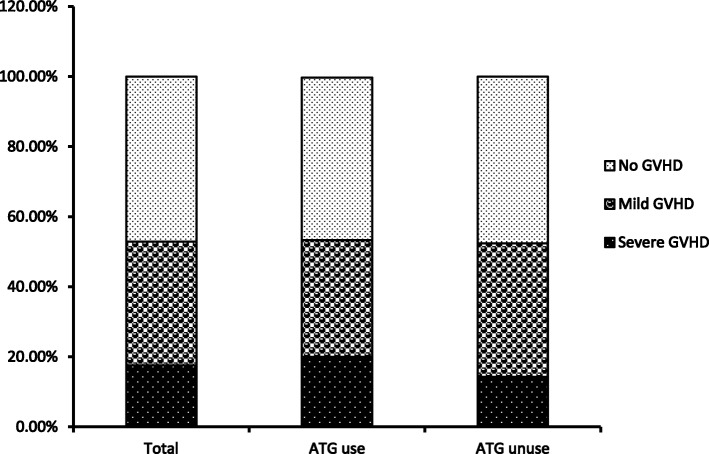
GVHD property in ATG used group and ATG unused group. T test analysis indicated there was no statistic differences between each group in each grade

Patients enrolled in this study had a total GVHD occurrence of 52.9% (27/51). In the ATG-treated group, the incidence of GVHD was 53.3% (16/30) which included 6 patients with lethal GVHD (20%) and 10 surviving patients from GVHD (33.3%). In the control group, the incidence of GVHD was 52.3% (11/21), which included 3 patients with lethal GVHD (14.3% incidence, 3/21), 8 patients with mild GVHD, and the remaining 10 patients did not develop GVHD. Statistical analysis showed no significant difference between the grades of GVHD in the two groups (Fig. 1).

### GVHD severity and different lymphocyte subsets

CD3-, CD4-, CD8-T cells, NK cells (CD3-CD56+), activated CD4+ T cells (CD4 + CD25+) cells were analyzed each month after transplantation. Retrospective study was used to collect the time point before GVHD happened in patients diagnosed with aGVHD. In patients without GVHD, data from 3 months post- transplantation were used for analysis.

**Fig. 2:**
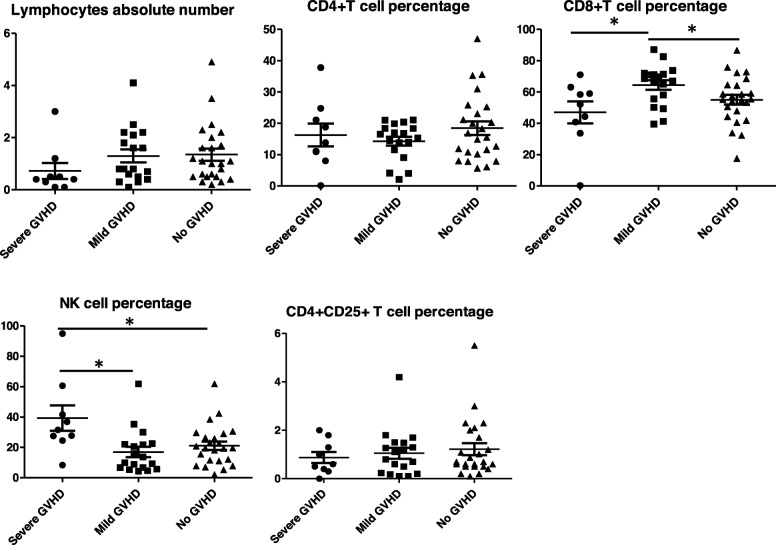
lymphocytes and T cell subsets percentage at the time point of GVHD happened in those GVHD suffering patients, for those without GVHD, 3 months after transplantation were used to collect clinical data. * *p* < 0.05

**Fig. 3:**
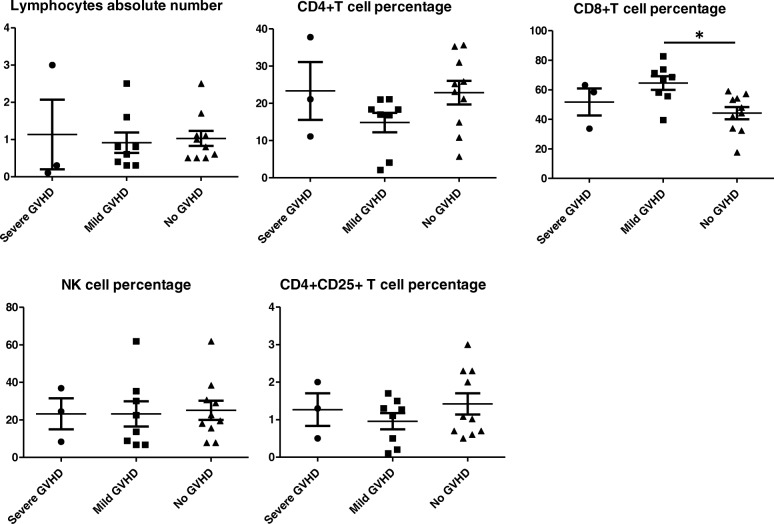
lymphocytes and T cell subsets percentage in ATG unused group at the time point of GVHD happened in those GVHD suffering patients, for those without GVHD, 3 months after transplantation were used to collect clinical data. * *p* < 0.05

**Fig. 4:**
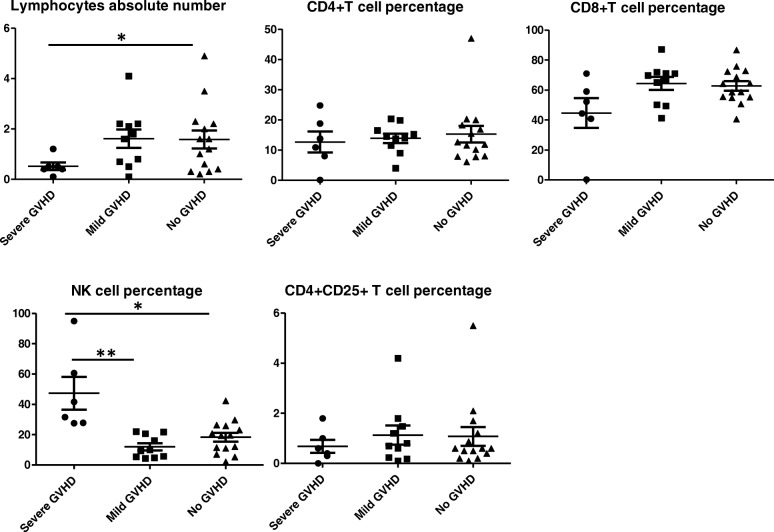
lymphocytes and T cell subsets percentage in ATG used group at the time point of GVHD happened in those GVHD suffering patients, for those without GVHD, 3 months after transplantation were used to collect clinical data. * *p* < 0.05, ** *p* < 0.01

In order to predict GVHD development, T cell subsets were compared within different grades of GVHD. Results showed that NK Cell percentage were noticeably different in ATG used group: in severe GVHD is 47.34%, in mild GVHD group was11.98%, while in no GVHD group is 18.3%. However, in the control group, data indicated the median NK cell percentage in severe GVHD is 23.27, 23.22% in mild GVHD group, and 21.13% in the group without GVHD. While there was no difference across different grades of GVHD in the absolute cell counts for CD4+ T cell subsets, CD4 + CD25 + T cell subsets, NK cell appeared to be a useful probe to indicate the severity of GVHD. However, there was some difference between the severe GVHD and mild GVHD groups in terms of CD8 T cell and NK cell subsets. In NK cell subsets, there was difference between severe GVHD and group without GVHD, although there was no difference between mild GVHD and group without GVHD. In terms of the CD8 T cell subset, there was difference between mild GVHD and group without GVHD while no difference was found between severe GVHD and no GVHD (Fig. 2). Because ATG play an important role in GVHD prevention, we compared the absolute lymphocyte number of various T cell subsets in the ATG unused group and ATG used group. in ATG unused group, there was no difference between severe GVHD and no GVHD groups when comparing absolute lymphocytes number, NK cell percentage, CD4+ T cell percentage and Treg cell percentage (Fig. 3), only the percentage of CD8+ T cells was significantly different between mild GVHD group and no GVHD group. However, In ATG used group, patients suffered from severe GVHD have lower absolute lymphocytes number and higher NK cell percentage compared with mild or no GVHD patients (Fig. 4).

## Discussion

Furthermore, we analyzed T cell and NK cell functions, T cell subsets including CD4+ T cells, CD8+ T cells and CD4 + CD25 + T cells. Our data showed that NK cells were correlated with GVHD occurrence in the ATG group while no correlation found between NK cells and GVHD in the control group. In addition, although our results indicate that GVHD incident was slightly higher in the ATG group, there was no statistical significance.

The standard GVHD grading system was proposed by Dr. Tomas [14]. However, the differences in grading are not always correlated with the prognosis, which may in fact be more closely associated with the immune status of patients. However, we can take advantage of the trend that severe GVHD are typically lethal while mild GVHD can be much better managed. Our study used retrospective analysis to separate severe GVHD from mild GVHD by using changes in the NK cell percentage to indicate the incidence of GVHD before the onset of clinical symptoms and begin early intervention to reduce the progression of severe GVHD.

In this study, percentage instead of absolute count number was used to analysis lymphocyte subsets. Due to the different condition regimens, T cell repertoire recovery can vary. In addition, the absolute T cell number recovery period were different, which cannot be used to predict T cell subset recovery. However, during T cell reconstitution, the percentage of different T cell subsets change proportionally, hence different T cell subsets can be compared this way.

The optimal balance between GVHD and GVL appears to be an excellent parameter to achieve long-term survival after allo-genetic stem cell transplantation. Previous study has showed that NK cells can eliminate leukemic cells, restore immunity early after stem cell transplant, while at the same time minimal graft versus host disease [15]. The use of a novel composite end point called GVHD-free progression-free survival (GPFS) was initiated in 2016 by Dr.Malard and colleagues. GPFS was defined as a combination of survival with no evidence of relapse progression, grade III to IV acute GVHD (aGVHD), and systemic therapy–requiring chronic GVHD (cGVHD). Their study indicated that NKT cells in the graft were associated with improved progression-free survival [16]. Other studies in patients that received allo-SCT treatment also showed that higher NK cell dose in donor graft associated with lower risk of relapse and long-term primary disease remission [17, 18]. The benefit from NK cell may be attributed to its introduction of controllable GVHD together with an optimal degree of GVL effect, In a separate study, it was shown that serum IFN-γ increased along with the expansion of NKT-like cells in GVHD patients, and patients benefitted through treatment with basiliximab (anti-CD25 antibody) and etanercept (anti- IFN-γ antibody) [3]. Kim et al. revealed that lower frequencies of iNKT cells (invariant NKT cells) were associated with higher GVHD incidence [19]. The study of NK cells showed similar suppression function on GVHD [20]. However, these data were based on NK or NKT cells before transfused into recipients. on the one hand, how the transferred NKT cells proliferation and survive after transplantation were unclear. On the other hand, NK or NKT cells homostasis after allo-SCT was unknown. Our study showed that NK cells proliferated post-transplant, which was different from previously-existing NK cells in graft.

NK cells are an important arm of the innate immune system and play an important role in mediating anti-tumor immunity of the recipient. Compared with conventional T cells, NK cells have many advantages. For example, allogeneic NK cells can be used as effector cells without the requirement for HLA matching and they do not cause GVHD [21].

Previous study showed an increase in NK cells may induce mild to middle degree of GVHD, if clinicians can intervene at the optimal time point to control GVHD, patients may benefit from the intervention. However, exact higher cell number may connect with poor prognosis of GVHD. NKT cell subsets may have different function in GVHD producing. Hu et al. showed that in GVHD patients, NKG2A + subset was decreased compared to control subsets. However, during recovery from GVHD, the number of NKG2A increased [22]. Monitoring the number of NKG2A provide clues for GVHD intervention and treatment. By adding ATG to the pretreatment protocol, we demonstrated that the percentage of NK cells is negative correlated with GVHD severity. However, to what extent that the addition of ATG in pretreatment regimen influences GVHD development requires further study.

## Conclusion

Our study showed that ATG can prevent GVHD by increase NK cell percentage. Monitoring the change of NK cell percentage after allogeneic stem cell transplantation can predict the severity of GVHD in the ATG used group. Our study also indicated in the control group, there was no significant difference between severe GVHD and mild GVHD in terms of NK cell percentage. Furthermore, other T cell subsets including CD4 T cells, CD8 T cells, Tregs, and CD8 + CD38+ T cells were unable to indicate GVHD incidence. The dynamic monitoring of lymphocyte subsets in post-transplant patients using ATG as a pretreatment scheme, especially the monitoring of NK cells, can be of great significance in clinic, and the occurrence can be predicted before the clinical occurrence of lethal GVHD symptoms, thus reducing the mortality rate of after transplantation.

## Supplementary information

**Additional file 1: Figure S1.** NK cells were defined as CD3-CD56 + CD16+, Treg cells were defined as CD4 + CD25 +.

## Data Availability

The datasets used and analyzed during the current study are available from the corresponding author on reasonable request.

## Abbreviations

AA: Aplastic anemia
aGVHD: Acute GVHD
ALL: Acute lymphocyte leukemia
allo-HCT: Allo-genetic hematopoietic stem cell transplantation
AML: Acute myeloid leukemia
ATG: Antithymocyte globulin
cGVHD: Chronic GVHD
CML: Chronic myeloid leukemia
GVHD: GFraft-versus-host disease
MDS: Myelodysplastic syndrome
PNH: Paroxysmal nocturnal hemoglobinuria
