# Supplementary figures and images for: NK cell predicts the severity of acute graft-versus-host disease in patients after allogeneic stem cell transplantation using antithymocyte globulin (ATG) in pretreatment scheme
Source: BMC Immunol. 2019 Dec 9;20:46. doi: 10.1186/s12865-019-0326-8 (PMC6902350; doi:10.1186/s12865-019-0326-8)

Supplemental Fig.1

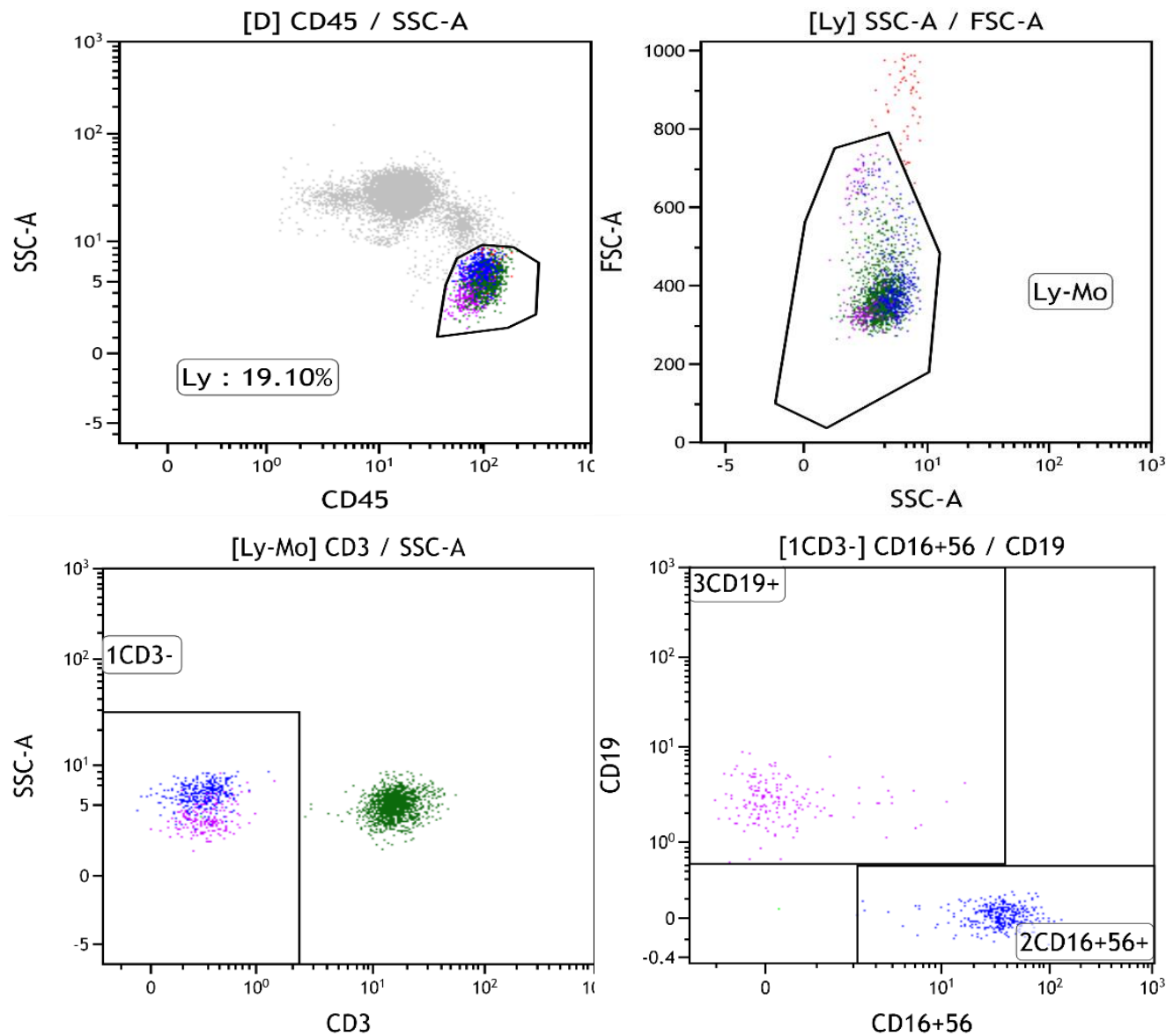

Supplement: Supplementary file 1 — Additional file 1: Figure S1. NK cells were defined as CD3-CD56 + CD16+, Treg cells were defined as CD4 + CD25 +. [file 12865_2019_326_MOESM1_ESM.pdf]
